# Oral intake of Heat-Killed *Lactiplantibacillus plantarum* Alleviates Bone Loss in an Ovariectomized Mouse Model Similarly to Live *L. plantarum*

**DOI:** 10.4014/jmb.2510.10013

**Published:** 2026-01-13

**Authors:** Yeonjin Lim, Ok-Jin Park, Chaeyeon Park, Bo-Min Kim, Cheol-Heui Yun, Seung Hyun Han

**Affiliations:** 1 Department of Oral Microbiology and Immunology, and DRI, School of Dentistry, Seoul National University, Seoul 08826, Republic of Korea; 2 Department of Agricultural Biotechnology, and Research Institute for Agriculture and Life Sciences, Seoul National University, Seoul 08826, Republic of Korea; 3 Institute of Green Bio Science Technology, Seoul National University, Pyeongchang 25354, Republic of Korea

**Keywords:** *Lactiplantibacillus plantarum*, Postbiotics, Probiotics, Osteoporosis

## Abstract

Probiotics, including *Lactiplantibacillus plantarum*, have therapeutic potential to alleviate osteoporosis, which is particularly common in postmenopausal women with increasing bone fracture risk. Since live probiotics may cause adverse effects under certain conditions, such as in immunocompromised individuals, postbiotics, could be a safer alternative. In this study, we investigated whether heat-killed *L. plantarum* KCTC 10887BP (K-Lp, postbiotic form) has comparable therapeutic effects to live *L. plantarum* KCTC 10887BP (L-Lp, probiotic form) on bone loss in a mouse postmenopausal osteoporosis model. Oral administration of either L-Lp or K-Lp significantly improved bone parameters, including trabecular bone volume, thickness, and number, compared to ovariectomy (OVX) control mice. Both treatments significantly increased bone mass and bone mineral density, elevated serum procollagen 1 N-terminal propeptide levels as a marker of bone formation, and reduced the number of tartrate-resistant acid phosphatase-positive osteoclasts in femoral tissue. Furthermore, oral administration with L-LP or K-Lp increased runt-related transcription factor 2, alkaline phosphatase, and collagen type I alpha 1 chain, which are key markers for osteoblast differentiation and activation. However, no effects were observed in Sham mice. Either L-Lp or K-Lp demonstrated similar therapeutic effects against bone loss in a postmenopausal mouse model. These findings suggest that heat-killed *L. plantarum* KCTC 10887BP exhibited comparable effects to live bacteria in this OVX model, and that postbiotics could serve as a therapeutic alternative for osteoporotic bone loss.

## Introduction

Bone health is essential for overall well-being as it provides structural support, protects vital organs, and plays a critical role in mineral homeostasis. Osteoblasts, which are responsible for bone formation, synthesize extracellular proteins that are important for bone mineralization, such as collagen type I alpha 1 (Col1a1). Runt-related transcription factor 2 (Runx2) plays an indispensable role in the proper function of mature osteoblasts, including the production of bone matrix [1]. Meanwhile, osteoclasts play a pivotal role in bone resorption, and a histochemical marker of osteoclasts is tartrate-resistant acid phosphatase (TRAP) [2]. The dynamic process of bone metabolism involves a fine balance between osteoblast and osteoclast functions. When this balance is disrupted and bone resorption outpaces bone formation, it can cause osteoporosis, or reduced bone mineral density that results in a higher risk of fractures [3]. This disease is especially prevalent in postmenopausal women as well as the elderly, and its incidence continues to rise as human life expectancy increases [4]. Consequently, osteoporosis not only severely impairs quality of life but also places a substantial economic burden on healthcare systems worldwide [5]. Therefore, development of effective therapeutic approaches for osteoporosis remains an essential research priority.

Probiotics are live microorganisms that, when administered in adequate amounts, confer health benefits on the host by modulating the gut microbiota and interacting with the host’s immune and metabolic systems [6]. *Lactobacillus* species are well-studied probiotic strains renowned for their ability to modulate the immune response, promote gastrointestinal health, and exert anti-inflammatory effects [7-9]. Among the *Lactobacillus* species, *Lactiplantibacillus plantarum* KCTC 10887BP (also known as *L. plantarum* K8) is a kimchi-derived strain. Several studies have shown that lysates of *L. plantarum* KCTC 10887BP can regulate immune response [10]. Additionally, many *Lactobacillus* species can improve bone health. For example, *Lactobacillus rhamnosus* GG and *L. brevis* AR281 ameliorate osteoporosis in an ovariectomized animal model [11, 12]. Also, Kim *et al*. demonstrated that an extract of *L. paracasei* L30 stimulates the osteoblast differentiation of human bone marrow mesenchymal stem cells *in vitro* [13]. A clinical trial by Jansson *et al*. demonstrated that administration of probiotics consisting of a mixture of three Lactobacilli strains to healthy postmenopausal women offered protection against lumbar spine bone loss [14]. These experiments suggest that probiotics can serve as a therapeutic agent for osteoporosis.

While probiotics are generally considered safe, they may pose risks for immunocompromised populations such as patients, the elderly, and young children. For instance, after the administration of live probiotics, a patient developed sepsis [15]; an immunocompromised patient suffered from systemic fungemia [16]; and *Lactobacillus* spp. bacteremia has also been reported [17]. These adverse effects may arise mostly due to the live nature of probiotics. To minimize these adverse effects, postbiotics, which are defined as a “preparation of inanimate microorganisms and/or their components that confers a health benefit on the host” [18], can also be used. Aside from safety, postbiotics offer additional advantages over probiotics, such as being easily transportable and storable, precise, and expedient in production technology, and quantitative control. Moreover, their exceptional processing capabilities enable their application across various products including jelly, beverages, health functional foods, and cosmetics [19]. Due to these advantageous attributes, the utilization of postbiotics is progressively expanding.

In previous studies, postbiotics have been shown to impact bone health by modulating immune responses and promoting calcium absorption [20]. Postbiotics offer advantages in safety, utility, management, and maintenance over probiotics. Therefore, if postbiotics demonstrate comparable effects on bone metabolism, they could be a preferable alternative to probiotics. However, studies comparing the efficacy of probiotics versus postbiotics in attenuating bone loss are yet to be conducted. In this study, we investigated whether heat-killed *L. plantarum* KCTC 10887BP (K-Lp, postbiotic form) exhibits similar effects to live *L. plantarum* KCTC 10887BP (L-Lp, probiotic form) in the treatment of bone loss using an ovariectomy (OVX)-induced osteoporosis animal model that mimics postmenopausal osteoporosis in humans.

## Materials and Methods

### Materials

TRAP staining kit was obtained from Sigma-Aldrich Inc. (USA). Mayer’s Hematoxylin histological staining reagent was purchased from Dako (Glostrup, Denmark). Eosin Y solution was obtained from BBC Biochemical (USA). Xylene substitute was purchased from Labcore (USA). Ethylenediaminetetraacetic acid (EDTA) was obtained from Hyclone (USA). The antibodies specifically binding to Runx2, Col1a1, and Hoechst 33342 were acquired from Santa Cruz Biotechnology (USA). Anti-alkaline phosphatase (ALP) antibody was purchased from Abcam (UK). Formalin was obtained from Biosesang (Republic of Korea).

### Bacteria Preparations

*Lactiplantibacillus plantarum* KCTC 10887BP was acquired from the Korean Collection for Type Culture (Republic of Korea). The bacteria were grown in MRS broth (Difco Laboratories) for 24 h at 37°C under static conditions. On the following day, it was sub-cultured into fresh MRS and grown until the log phase. Then the bacteria were pelleted through centrifugation at 6,150 × *g* for 10 min at 4°C and subsequently resuspended in phosphate buffered saline (PBS). After determining colony-forming unit (CFU)-equivalents, the harvested *L. plantarum* was divided into two parts. One part was heat-killed by incubation at 70°C for 2 h [21, 22], while the other part was pelleted and resuspended in PBS to maintain live cells. To verify the inactivation of the K-Lp stocks, they were plated onto MRS agar plates and incubated for 24 h to evaluate the absence of *L. plantarum*. Furthermore, the L-Lp stocks were cultured on MRS agar plates to confirm their viability and maintain their CFUs.

### Animals

Approval for all animal experiments was obtained from the Institutional Animal Care and Use Committee of Seoul National University (SNU-221004-3). All animal studies were performed in accordance with the ARRIVE guidelines. Eleven-week-old female C57BL/6 mice were obtained from DooYeol Biotech (Republic of Korea). The animals were kept in a controlled environment with a temperature range of 22-24°C, humidity of 55%, and a 12 h light-dark cycle within a specific pathogen-free facility. Bilateral ovariectomy was conducted on 12-week-old female C57BL/6 mice following established protocols [23]. Sham-operated (Sham) mice underwent an identical surgical procedure to the OVX mice but without ovary removal. At three weeks post-surgery, animals were randomly allocated into three experimental groups: Sham-operated, OVX with PBS as control, and OVX with K-Lp or L-Lp treatment. The mice were administered with 8 × 10^10^ CFU/kg of L-Lp or K-Lp three times weekly for four weeks. The Sham group was administered PBS as a vehicle control.

### Micro-Computed Tomography (micro-CT)

OVX mice were administered 8 × 10^10^ CFU/kg of L-Lp or K-Lp three times per week for four weeks. Then, the mice were sacrificed, and the right femurs were removed and fixed in 10% formalin. Fixed femurs were scanned using X-ray micro-CT (Skyscan 1272 scanner; Skyscan, Belgium) at 70 kV, 142 mA, 10 W, 0.5 mm aluminum filter, and 10 μm per pixel scan resolution. The images were reconstructed using the programs NRecon (Skyscan, Version 1.7.3.1) and Data Viewer (Skyscan, Version 1.5.6.2). Then, it was analyzed using the software CT Analyzer (Skyscan, Version 1.17.7.2). Three-dimensional (3D) volume of interest images were generated using the CTvol software program (Skyscan, Version 2.3.2.0). For the quantitative assessment of trabecular bone, we selected the ROI from 0.5 mm above the growth plate and analyzed 1.3 mm of the trabecular bone region. The trabecular bone parameters, including bone volume fraction (BV/TV), trabecular thickness (Tb.Th), trabecular number (Tb.N), and trabecular separation (Tb.Sp), were obtained through voxel-based 3D reconstruction of micro-CT images.

### Histology, Hematoxylin and Eosin (H&E) Staining, TRAP Staining, and Immunohistochemistry

After micro-CT scanning, femurs were decalcified in 10% EDTA in PBS for a week at 4°C with agitation. After decalcification, the femurs were embedded in paraffin, longitudinally sectioned at a thickness of 5 μm, and affixed to glass slides. The paraffin-embedded femur sections were then subjected to staining using H&E or TRAP, as described previously [23]. Osteomeasure software was used to measure osteoclast surfce per bone surface (Oc.S/BS) in TRAP-stained femur sections. For each sample, three non-overlapping fields within the trabecular region of the distal femur were analyzed, and values were normalized to bone surface (BS). For immunohistochemical analysis of bone formation markers, we used mouse anti-Runx2 monoclonal antibody, mouse anti-ALP monoclonal antibody, and rabbit anti-Col1a1 antibody, visualized using EnVision Detection systems with peroxidase-diaminobenzidine (DAKO, UK Ltd., UK). The images were collected using a confocal microscope with a digital camera system (BX-51 with DP72; Olympus, Japan). Mean fluorescence intensity (MFI) was measured using ZEN 3.1 Blue Edition software (Carl Zeiss GmbH, Germany). Quantification was performed on one anatomically matched region near the growth plate per section, and MFI values were averaged across samples within each group.

### Enzyme-Linked Immunosorbent Assay (ELISA)

The P1NP (Procollagen 1 N-terminal propeptide) levels were measured using mouse PINP ELISA Kit (Elabscience, China) according to the manufacturer’s instructions.

### Statistical Analysis

All experiments were meticulously conducted, each repeated three to five times, and the resulting data were consistently expressed as the mean ± standard deviation (SD) of triplicate samples. Statistical significance was determined using one-way ANOVA, conducted with GraphPad Prism 6 software (GraphPad Software Inc., USA). Significant differences compared to the control group are indicated by asterisks (*), with a threshold of *p* < 0.05.

## Results

### Oral Administration of Heat-Killed *L. plantarum* Results in a Comparable Increase in Trabecular Bone Volume to Live *L. plantarum*

**Fig. 1:**
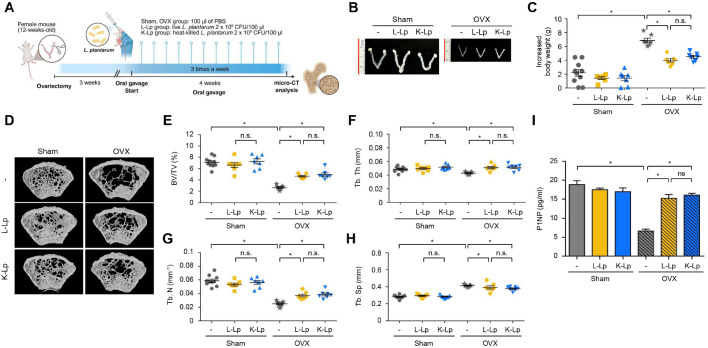
Oral administration of heat-killed *L. plantarum* increases trabecular bone volume comparably to live *L. plantarum*. (**A**) Experimental scheme. *L. plantarum* was cultured, harvested, and CFU was determined. The bacteria were then prepared as live cells or heat-killed by incubation at 70°C for 2 h. Twelve-week-old female C57BL/6 mice (n ≥ 7) were ovariectomized or sham-operated. Three weeks post-surgery, mice were administered with PBS or 8 × 10^10^ CFU/kg of L-Lp or K-Lp three times per week for four weeks by oral gavage. (**B**) Uterus weight was measured and photographed at the end of the treatment. (**C**) Body weight of mice was assessed at weeks 12 and 19, and the change over time was calculated. (**D**) Three-dimensional images of the femurs were obtained using the micro- CT analyzer. (**E-H**) Trabecular bone volume per total bone volume (BV/TV) (**E**), trabecular thickness (Tb.Th) (**F**), trabecular number (Tb.N) (**G**), and trabecular separation (Tb.Sp) (**H**) of mouse femurs were analyzed. (**I**) Serum P1NP levels were measured using an ELISA assay. n.s.: no significant difference; *p < 0.05.

To assess the effects of L-Lp and K-Lp on postmenopausal osteoporosis, mice underwent either OVX or Sham. At three weeks post-surgery, Sham control and OVX mice were randomly allocated into three treatment groups: L-Lp, K-Lp, or PBS. Treatments were administered via oral gavage three times weekly for a duration of four weeks (Fig. 1A). The mice exhibited a decrease in uterus size (Fig. 1B), while body weight increased (Fig. 1C). Micro-CT scans of the femurs demonstrated a significant reduction in trabecular bone volume (BV/TV) in OVX control mice compared to Sham mice (Fig. 1D-1E). While there were no changes observed when L-Lp or K-Lp were administered to Sham mice, when administered to OVX mice, both L-Lp and K-Lp yielded a marked increase in BV/TV, Tb.Th, and Tb.N compared to those administered PBS (Fig. 1E-1G). Tb.Sp increased in OVX mice compared to Sham mice, and this parameter remained unchanged with the administration of either L-Lp or K-Lp (Fig. 1H). In addition, oral administration of L-Lp or K-Lp to OVX mice resulted in elevated serum P1NP levels, indicating an upregulation of bone formation (Fig. 1I). These findings suggest that K-Lp has equivalent effects to L-Lp in enhancing bone volume in OVX mice.

### Oral Administration of Heat-Killed *L. plantarum* Induces Histological Changes Similar to Live *L. plantarum*

**Fig. 2:**
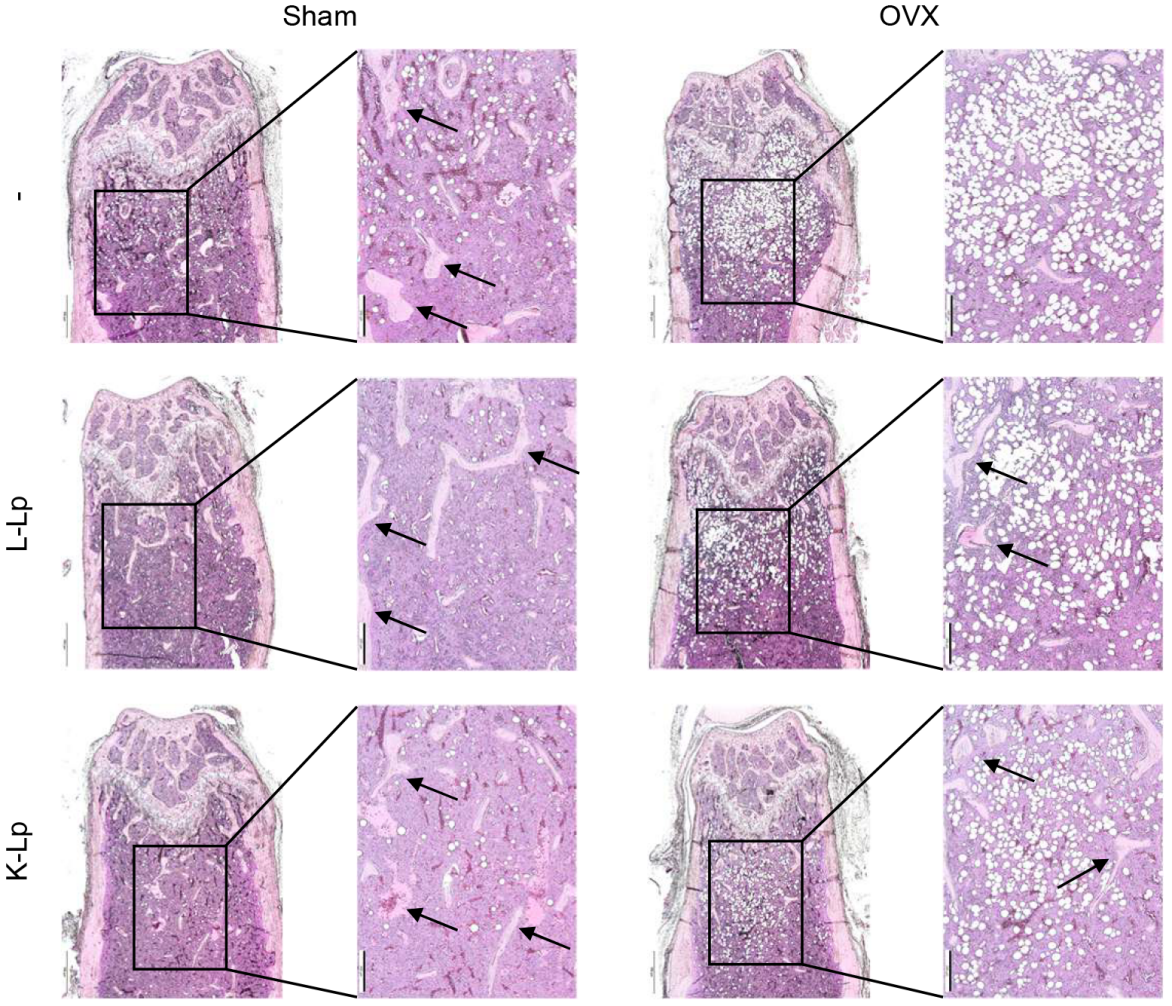
Heat-killed *L. plantarum* induces histological changes similar to live *L. plantarum*. Sham and ovariectomized mice were administered PBS or 8 × 10^10^ CFU/kg of L-Lp or K-Lp three times weekly for four weeks via oral gavage. Femurs were decalcified, embedded in paraffin, and sectioned for H&E staining to assess general morphology. Representative images from one of three similar experiments are shown. Scale bars: 500 μm (left panels); 200 μm (right panels).

H&E staining was conducted on femur paraffin sections to evaluate histomorphological changes in the distal femurs. Consistent with the micro-CT results, the H&E staining results indicated a remarkable reduction in trabecular bone density in the OVX control group compared to the Sham group. Administration of either L-Lp or K-Lp to OVX mice effectively prevented trabecular bone loss. Additionally, we observed that either L-Lp or K-Lp effectively suppressed bone marrow adiposity, indicating a shift in mesenchymal stem cell fate from adipogenesis to osteogenesis (Fig. 2).

### Oral Administration of Heat-Killed *L. plantarum* Inhibits OVX-Induced Osteoclast Activity to a Similar Extent as Live *L. plantarum*

**Fig. 3:**
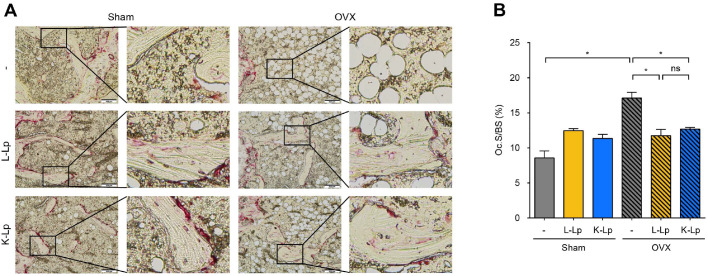
Heat-killed *L. plantarum* attenuates OVX-induced osteoclast activity similarly to live *L. plantarum*. Sham-operated and ovariectomized mice were orally administered either PBS or 8 × 10^10^ CFU/kg of L-Lp or K-Lp by gavage three times per week for four weeks. Femurs were decalcified and embedded in paraffin. (**A**) Femur paraffin sections were subjected to TRAP staining to assess osteoclast activity. Scale bars: 200 μm. One of three similar results is shown. (**B**) Histomorphometric analysis was performed for parameters related to bone resorption. OC.S/BS, osteoclast surface per bone surface. The data shown are the mean value ± SD of triplicate samples and are representative of three similar independent experiments. n.s.: no significant difference; *p < 0.05.

To examine the effects of L-Lp or K-Lp on osteoclast differentiation, TRAP staining was performed on femur sections. In Sham mice, treatment with L-Lp or K-Lp resulted in similar levels of TRAP-positive surface area and trabecular bone surface compared to those in the untreated group. OVX control mice exhibited reduced trabecular bone surface compared with Sham control mice, but administration of L-Lp or K-Lp ameliorated this reduction in OVX mice compared with OVX control mice (Fig. 3A). In the femur sections, we measured TRAP-positive areas on bone surfaces using the Image J program; such areas indicate osteoclast presence. Femur sections from OVX control mice exhibited a marked increase of approximately 8.54% in OC.S/BS compared to Sham control mice. Additionally, OVX mice administered L-Lp and K-Lp each showed a notable reduction in OC.S/BS by about 5.35% and 4.42%, respectively (Fig. 3B). These results indicate that both L-Lp and K-Lp alleviate osteoclast differentiation in an OVX-induced osteoporosis mouse model but do not affect healthy mice.

### Oral Administration of Heat-Killed *L. plantarum* in OVX Mice Enhances Runx2, ALP, and Col1a1 Expression to a Similar Extent as Live *L. plantarum*

**Fig. 4:**
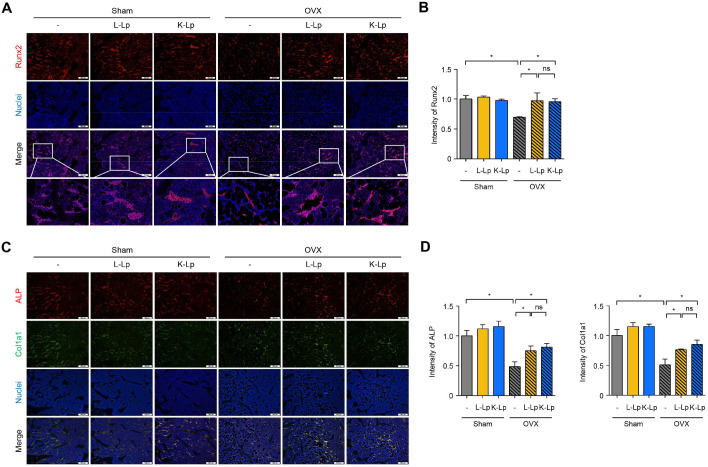
Heat-killed *L. plantarum* enhances Runx2, ALP, and Col1a1 expression in OVX mice similarly to live *L. plantarum*. Sham and ovariectomized mice were orally administered PBS or 8 × 10^10^ CFU/kg of L-Lp or K-Lp three times per week for four weeks. Subsequently, femurs were decalcified and embedded in paraffin. (**A, B**) The paraffin sections of the femur were subjected to immunofluorescence staining using antibody specific to Runx2 followed by staining with Hoechst 33342 to visualize nuclei. The merged fluorescence signal of Runx2 and nuclei in the images reflects nuclear localization of Runx2. Scale bars: 200 μm. One of three similar results is shown. (**C, D**) Bar charts present the fold-change of ALP, and Col1a1-positive areas relative to those of the OVX group. n.s.: no significant difference; *p < 0.05.

To investigate whether the effects of L-Lp and K-Lp on bone metabolism are associated with regulation of osteoblast differentiation, we conducted immunofluorescence staining on paraffin sections of femurs using an antibody specific to Runx2, ALP, and Col1a1. Runx2 is an essential transcription factor associated with osteoblast differentiation [24], and ALP is an enzyme pivotal for hydrolyzing phosphate esters during early matrix mineralization [25]. Col1a1 is a primary fibrillar protein of the bone extracellular matrix [26]. These markers exhibit elevated expression as osteoblast differentiation is upregulated. As shown in Fig. 4A-4D, the femur sections from Sham mice administered with L-Lp and K-Lp exhibited no changes in the intensity of Runx2, ALP, and Col1a1 compared to the Sham control group. In contrast, the intensity of Runx2, ALP, and Col1a1 was diminished in OVX control mice but recovered in OVX mice that received either L-Lp or K-Lp. Especially, Runx2 (red) and Hoechst 33342 (blue) signals overlap in the nuclei, resulting in a pink merged signal. This colocalization indicates nuclear localization of Runx2.These results suggest that both L-Lp and K-Lp attenuate bone loss by upregulating osteoblast activity in the OVX mouse model.

## Discussion

Postbiotics exhibit enhanced physicochemical stability during processing and storage, require simpler production, and eliminate the risk of microbial translocation associated with living probiotics. Therefore, if postbiotics confer effects comparable to those of probiotics, the use of postbiotics would be advantageous. The results of our study demonstrate that oral administration of either L-Lp or K-Lp increased bone volume to a similar extent in an OVX mouse model mimicking postmenopausal osteoporosis in humans, while showing no effect in the Sham control mouse model. The alleviation effect might be due to the inhibition of osteoclast differentiation and promotion of osteoblast differentiation by both L-Lp and K-Lp. Importantly, our study is the first to demonstrate that heat-killed *L. plantarum* KCTC 10887BP (postbiotic form) exhibits bone-protective effects comparable to its live form (probiotic form) form in an OVX mouse model, highlighting the potential of postbiotic strategies for osteoporosis management.

Both L-Lp and K-Lp exhibited the same ability to attenuate bone loss in the osteoporosis mouse model. Analysis of TRAP-positive surface areas in femur sections revealed a decrease in both the L-Lp and K-Lp groups compared to the control group, along with an increased expression of Runx2 in femur sections of both groups. Live *L. plantarum* HFY15 has been shown to prevent retinoic acid-induced secondary osteoporosis in rats, with induction levels comparable to zoledronic acid, a pharmacologic treatment for osteoporosis [27]. Based on these findings, it is reasonable to expect that *L. plantarum* KCTC 10887BP used in our study may exert similar bone-protective effects. These observations are highly significant as they highlight the potential therapeutic efficacy of postbiotics in treating osteoporosis, achieved through the promotion of osteoblast-mediated bone formation with the inhibition of osteoclast differentiation. Several other studies also suggested that both live and heat-killed Lactobacilli spp. exhibit comparable effects on various injuries. Live and heat-killed *L. reuteri* ameliorated alveolar bone loss of ligature-induced periodontitis [28]. Additionally, both live and heat-killed *L. plantarum* CCFM639 alleviated chronic aluminum-induced brain and liver injuries [29]. Based on these results, it can be inferred that heat-killed probiotics effectively modulate immune responses. This not only enhances our understanding of the potential applications of postbiotics, but also reinforces their validity as a promising method for the management of osteoporosis.

We observed that both L-Lp and K-Lp increased bone volume in a postmenopausal osteoporosis mouse model, indicating their potential in alleviating OVX-induced bone loss by inhibiting osteoclast differentiation and promoting osteoblast differentiation in femur trabecular bone. In previous studies, probiotics have consistently demonstrated the ability to enhance osteoblast differentiation while reducing osteoclast differentiation, thus showing promise in attenuating osteoporosis. For instance, Yang *et al*. showed that *L. plantarum* GKM3 and *Lacticaseibacillus paracasei* GKS6 effectively ameliorated bone loss in OVX mice by promoting osteoblast differentiation and inhibiting osteoclast differentiation [30]. Similarly, Lee & Kim reported that *L. plantarum* A41 and *Limosilactobacillus fermentum* SRK414 upregulated the mRNA expression of osteoblast differentiation markers [31]. Moreover, there are several studies have shown that heat-inactivated *Lactobacillus casei* strain GKC1 mitigates osteoporosis [32], and that killed *Lacticaseibacillus paracasei* GMNL-653 exhibits antiosteoporotic activity [33]. Myeong *et al*. found that the postbiotic *L. plantarum* MD35 reduced osteoclast differentiation by decreasing the expression of osteoclastogenic genes [34]. Collectively, these findings suggest that both live and heat-killed *Lactobacillus* modulate the balance between osteoblasts and osteoclasts, contributing to the attenuation of bone loss in estrogen-deficient osteoporosis in mice. Furthermore, these findings suggest that heat-stable cell wall components of *Lactobacillus* spp. might be a key factor in these osteoprotective effects, as evidenced by the comparable efficacy of both live and heat-killed bacteria. Although this study focused on a single strain, *L. plantarum* KCTC 10887BP, further comparative research involving other *L. plantarum* strains is needed to determine whether the observed bone-protective properties are unique to this strain or represent a broader characteristic of the species.

The comparable effects observed between postbiotics and probiotics can be attributed to the presence of heat-stable molecules in *L. plantarum* KCTC 10887BP. Previous studies have identified peptidoglycan (PGN) and lipoteichoic acid (LTA) as potential major factors contributing to the mitigating effect on osteoporosis. PGN, an essential component of bacterial cell walls, especially in Gram-positive bacteria where it comprises approximately 90%, has been implicated in modulating immune responses through released fragments in the bloodstream originating from the gut microbiota [35]. Upon breakdown in the host, PGN yields various fragments, including muramyl dipeptide (MDP), which activates nucleotide-binding oligomerization domain-containing protein 2. In a previous study, Park *et al*. demonstrated that MDP could augment bone mass by promoting the expression of osteoblast differentiation transcription factors [36, 23]. Additionally, LTA has also shown potential in attenuating osteoporosis by inhibiting osteoclast differentiation through interference with gelsolin-actin dissociation [37]. Also, Yang *et al*. showed that *Enterococcus faecalis* LTA inhibits macrophage differentiation toward osteoclasts [38]. Both PGN and LTA are heat-stable components of the bacterial cell wall [39] [40]. Although previous studies have demonstrated that purified bacterial components such as MDP and LTA can regulate osteoblast and osteoclast activity, these findings should be regarded as theoretical context rather than direct mechanistic evidence for *L. plantarum* KCTC 10887BP. In our study, both live and heat-killed *L. plantarum* increased bone mass in OVX mice, suggesting that cell wall components may underlie this effect. Accordingly, the osteoprotective activity observed here may be attributed to heat-stable molecules present in *L. plantarum* KCTC 10887BP.

Our results showed that both L-Lp and K-Lp had no marked effects on bone metabolism in sham-operated mice. This data is consistent with a previous study that administered *L. paracasei* or a mixture of three *Lactobacillus* strains in drinking water to 10-week-old female Sham control mice for 6 weeks, revealing no differences compared to the Sham control mice [41]. Also, this previous study demonstrated that *L. reuteri* ingestion led to a notable increase in bone formation rate in 14-week-old male mice but not in female mice [42]. It has been reported that administration of the yeast probiotic *Saccharomyces boulardii* reduced bone loss in OVX mice but exhibited no such impact in sham-operated mice [43]. Notably, the oral administration of L-Lp or K-Lp did not result in differences in healthy mice, indicating that the observed beneficial effects of probiotics may be limited to immunocompromised or osteoporotic conditions. This provides a scientific basis for considering probiotics and postbiotics as potential therapeutic agents for osteoporosis patients.

In this study, we demonstrated that probiotics and postbiotics exhibited comparable attenuation of bone loss caused by osteoporosis. This finding suggests that probiotics and postbiotics could serve as effective treatments for osteoporosis, with heat-stable components of probiotics acting as bone metabolism regulators. Notably, postbiotics have emerged as promising agents for safe application in immunocompromised patients due to their immunobiotic properties [44]. Moreover, they offer a cost-effective and more stable alternative to probiotics for managing osteoporosis. These findings open the way for further research into therapeutic methods targeting osteoporosis.

## References

[1] Long F (2011). Building strong bones: molecular regulation of the osteoblast lineage. Nat. Rev. Mol. Cell Biol..

[2] Teitelbaum SL (2000). Bone resorption by osteoclasts. Science.

[3] Tanaka Y, Nakayamada S, Okada Y (2005). Osteoblasts and osteoclasts in bone remodeling and inflammation. Curr. Drug Targets Inflamm. Allergy.

[4] Nih Consensus Development Panel on Osteoporosis Prevention D, Therapy. 2001. Osteoporosis prevention, diagnosis, and therapy. *JAMA* **285:** 785-795. https://doi.org/10.1001/jama.285.6.785. 10.1001/jama.285.6.785 11176917

[5] Burge R, Dawson-Hughes B, Solomon DH, Wong JB, King A, Tosteson A (2007). Incidence and economic burden of osteoporosis-related fractures in the United States, 2005-2025. J. Bone Miner. Res..

[6] Sanders ME. 2008. Probiotics: definition, sources, selection, and uses. *Clin Infect Dis.* **46 Suppl 2:** S58-61; discussion S144-151. https://doi.org/10.1086/523341. 10.1086/523341 18181724

[7] Choi JH, Moon CM, Shin TS, Kim EK, McDowell A, Jo MK (2020). *Lactobacillus paracasei*-derived extracellular vesicles attenuate the intestinal inflammatory response by augmenting the endoplasmic reticulum stress pathway. Exp. Mol. Med..

[8] Kang JH, Kang MS, Kim SD, Lee HK, Song SW, Nam CJ (2025). Single and repeated-dose toxicity studies by intravaginal administration of *Lactobacillus plantarum* ATG-K2 powder in female rats. Toxicol. Res..

[9] Yoo JW, Shin YJ, Ma X, Son YH, Jang HM, Lee CK (2022). The alleviation of gut microbiota-induced depression and colitis in mice by anti-inflammatory probiotics NK151, NK173, and NK175. Nutrients.

[10] Ahn YS, Park MY, Shin JH, Kim JY, Kwon O (2014). Lysate of probiotic *Lactobacillus plantarum* K8 modulate the mucosal inflammatory system in dextran sulfate sodium-induced colitic rats. Korean J. Food Sci. Anim. Resour..

[11] Guo M, Liu H, Yu Y, Zhu X, Xie H, Wei C (2023). *Lactobacillus rhamnosus* GG ameliorates osteoporosis in ovariectomized rats by regulating the Th17/Treg balance and gut microbiota structure. Gut Microbes.

[12] Yu J, Hang Y, Sun W, Wang G, Xiong Z, Ai L (2022). Anti-osteoporotic effect of *Lactobacillus brevis* AR281 in an ovariectomized mouse model mediated by inhibition of osteoclast differentiation. Biology (Basel).

[13] Kim I, Park S, Kim J, Park SY, Seo J, Roh S (2025). Treatment with *Lactobacillus paracasei* L30 extract induces osteogenic differentiation of human bone marrow mesenchymal stem cells in vitro. Biomed. Pharmacother..

[14] Jansson PA, Curiac D, Lazou Ahren I, Hansson F, Martinsson Niskanen T, Sjogren K (2019). Probiotic treatment using a mix of three *Lactobacillus* strains for lumbar spine bone loss in postmenopausal women: a randomised, double-blind, placebo-controlled, multicentre trial. Lancet Rheumatol..

[15] Kochan P, Chmielarczyk A, Szymaniak L, Brykczynski M, Galant K, Zych A (2011). *Lactobacillus rhamnosus* administration causes sepsis in a cardiosurgical patient--is the time right to revise probiotic safety guidelines. Clin. Microbiol Infect..

[16] Appel-da-Silva MC, Narvaez GA, Perez LRR, Drehmer L, Lewgoy J (2017). *Saccharomyces cerevisiae* var. *boulardii* fungemia following probiotic treatment. Med. Mycol. Case Rep..

[17] Kullar R, Goldstein EJC, Johnson S, McFarland LV (2023). *Lactobacillus* bacteremia and probiotics: a review. Microorganisms.

[18] Salminen S, Collado MC, Endo A, Hill C, Lebeer S, Quigley EMM (2021). The International Scientific Association of Probiotics and Prebiotics (ISAPP) consensus statement on the definition and scope of postbiotics. Nat. Rev. Gastroenterol. Hepatol..

[19] Zolkiewicz J, Marzec A, Ruszczynski M, Feleszko W (2020). Postbiotics-a Step beyond pre- and probiotics. Nutrients.

[20] Montazeri-Najafabady N, Ghasemi Y, Dabbaghmanesh MH, Ashoori Y, Talezadeh P, Koohpeyma F (2021). Exploring the bone sparing effects of postbiotics in the post-menopausal rat model. BMC Complement. Med. Ther..

[21] Lee CC, Liao YC, Lee MC, Cheng YC, Chiou SY, Lin JS (2022). Different impacts of heat-killed and viable *Lactiplantibacillus plantarum* TWK10 on exercise performance, fatigue, body composition, and gut microbiota in humans. Microorganisms.

[22] Kumar A, Saha MK, Kumar V, Bhattacharya A, Barge S, Mukherjee AK (2024). Heat-killed probiotic *Levilactobacillus brevis* MKAK9 and its exopolysaccharide promotelongevity by modulating aging hallmarks and enhancing immune responses in Caenorhabditis elegans. Immun. Ageing.

[23] Park OJ, Kwon Y, Kim J, Park C, Yun CH, Han SH (2023). Muramyl dipeptide alleviates estrogen deficiency-induced osteoporosis through canonical Wnt signaling. J. Pathol..

[24] Komori T (2006). Regulation of osteoblast differentiation by transcription factors. J. Cell. Biochem..

[25] Millan JL (2013). The role of phosphatases in the initiation of skeletal mineralization. Calcif. Tissue Int..

[26] Chen Y, Yang S, Lovisa S, Ambrose CG, McAndrews KM, Sugimoto H (2021). Type-I collagen produced by distinct fibroblast lineages reveals specific function during embryogenesis and Osteogenesis Imperfecta. Nat. Commun..

[27] Liu X, Zheng J, Li F, Yi R, Mu J, Tan F (2020). *Lactobacillus Plantarum* HFY15 helps prevent retinoic acid-induced secondary osteoporosis in wistar rats. Evid. Based Complement. Alternat. Med..

[28] Moraes RM, Lescura CM, Milhan NVM, Ribeiro JL, Silva FA, Anbinder AL (2020). Live and heat-killed *Lactobacillus reuteri* reduce alveolar bone loss on induced periodontitis in rats. Arch. Oral Biol..

[29] Tian F, Yu L, Zhai Q, Xiao Y, Shi Y, Jiang J (2017). The therapeutic protection of a living and dead *Lactobacillus* strain against aluminum-induced brain and liver injuries in C57BL/6 mice. PLoS One.

[30] Yang LC, Lin SW, Li IC, Chen YP, Tzu SY, Chou W (2020). *Lactobacillus plantarum* GKM3 and *Lactobacillus paracasei* GKS6 supplementation ameliorates bone loss in ovariectomized mice by promoting osteoblast differentiation and inhibiting osteoclast formation. Nutrients.

[31] Lee CS, Kim SH (2020). Anti-inflammatory and anti-osteoporotic potential of *Lactobacillus plantarum* A41 and *L. fermentum* SRK414 as probiotics. Probiotics Antimicrob. Proteins.

[32] Yang LC, Li TJ, Hu YF, Tsai YS, Wang CS, Lin SW (2025). Heat-inactivated *Lactobacillus casei* strain GKC1 Mitigates osteoporosis development *in vivo* via enhanced osteogenesis. Biochem. Biophys. Res. Commun..

[33] Jhong JH, Tsai WH, Yang LC, Chou CH, Lee TY, Yeh YT (2022). Heat-killed *Lacticaseibacillus paracasei* GMNL-653 exerts antiosteoporotic effects by restoring the gut microbiota dysbiosis in ovariectomized mice. Front. Nutr..

[34] Myeong JY, Jung HY, Chae HS, Cho HH, Kim DK, Jang YJ (2024). Protective effects of the postbiotic *Lactobacillus plantarum* MD35 on bone loss in an ovariectomized mice model. Probiotics Antimicrob. Proteins.

[35] Wolf AJ, Underhill DM (2018). Peptidoglycan recognition by the innate immune system. Nat. Rev. Immunol..

[36] Park OJ, Kim J, Yang J, Yun CH, Han SH (2017). Muramyl dipeptide, a shared structural motif of peptidoglycans, is a novel inducer of bone formation through induction of Runx2. J. Bone Miner. Res..

[37] Kwon Y, Yang J, Park OJ, Park C, Kim J, Lee D (2023). Lipoteichoic acid inhibits osteoclast differentiation and bone resorption via interruption of gelsolin-actin dissociation. J. Cell. Physiol..

[38] Yang J, Park OJ, Kim J, Baik JE, Yun CH, Han SH (2016). Lipoteichoic acid of *Enterococcus faecalis* inhibits the differentiation of macrophages into osteoclasts. J. Endod..

[39] Vollmer W, Blanot D, de Pedro MA (2008). Peptidoglycan structure and architecture. FEMS Microbiol. Rev..

[40] Park OJ, Yang J, Kim J, Yun CH, Han SH (2015). *Enterococcus faecalis* attenuates the differentiation of macrophages into osteoclasts. J. Endod..

[41] Ohlsson C, Engdahl C, Fak F, Andersson A, Windahl SH, Farman HH (2014). Probiotics protect mice from ovariectomy-induced cortical bone loss. PLoS One.

[42] McCabe LR, Irwin R, Schaefer L, Britton RA (2013). Probiotic use decreases intestinal inflammation and increases bone density in healthy male but not female mice. J. Cell. Physiol..

[43] Madel MB, Halper J, Ibanez L, Claire L, Rouleau M, Boutin A (2023). Specific targeting of inflammatory osteoclastogenesis by the probiotic yeast *S. boulardii* CNCM I-745 reduces bone loss in osteoporosis. Elife.

[44] Salva S, Tiscornia I, Gutierrez F, Alvarez S, Bollati-Fogolin M (2021). *Lactobacillus rhamnosus* postbiotic-induced immunomodulation as safer alternative to the use of live bacteria. Cytokine.

